# Ultrasound scan as a potential source of nosocomial and crossinfection: a literature review*

**DOI:** 10.1590/0100-3984.2014.0002

**Published:** 2015

**Authors:** André Hadyme Miyague, Fernando Marum Mauad, Wellington de Paula Martins, Augusto César Garcia Benedetti, Ana Elizabeth Gomes de Melo Tavares Ferreira, Francisco Mauad-Filho

**Affiliations:** 1 PhD, Coordinator of the Fetal Medicine Unit, Hospital Universitário Evangélico de Curitiba, Curitiba, PR, Brazil.; 2 PhD, MD, Sonographist, Faculdade de Tecnologia em Saúde (Fatesa), Ribeirão Preto, SP, Brazil.; 3 PhD, Department of Gynecology and Obstetrics, Faculdade de Medicina de Ribeirão Preto da Universidade de São Paulo (FMRP-USP), Ribeirão Preto, SP, Brazil.; 4 Masters, MDs, Sonographists, Faculdade de Tecnologia em Saúde (Fatesa), Ribeirão Preto, SP, Brazil.; 5 MD, Sonographist, Director, Faculdade de Tecnologia em Saúde (Fatesa), Ribeirão Preto, SP, Brazil.

**Keywords:** Ultrasonography, Transvaginal transducer, Cleaning, Disinfection

## Abstract

The authors review the main concepts regarding the importance of cleaning/disinfection of ultrasonography probes, aiming a better comprehension by practitioners and thus enabling strategies to establish a safe practice without compromising the quality of the examination and the operator productivity. In the context of biosafety, it is imperative to assume that contact with blood or body fluids represents a potential source of infection. Thus, in order to implement cleaning/disinfection practice, it is necessary to understand the principles of infection control, to consider the cost/benefit ratio of the measures to be implemented, and most importantly, to comprehend that such measures will not only benefit the health professional and the patient, but the society as a whole.

## Abstract (pt)

Os autores revisam os principais conceitos relativos à importância da limpeza/desinfecção das sondas ecográficas, visando sua melhor compreensão e possibilitando, assim, estabelecer estratégias para uma prática segura sem comprometer a qualidade do exame e a produtividade do operador. No contexto da biossegurança, é imperativo assumir que o contato com sangue ou fluidos corporais representa uma potencial fonte de infecção. Dessa forma, para que as práticas de limpeza/desinfecção possam ser instituídas, é necessário entender os princípios do controle de infecção, ponderar se os benefícios das medidas a serem instituídas são compatíveis com seus custos e consequências, e principalmente compreender que tais medidas não beneficiam apenas o profissional de saúde e o paciente, mas a sociedade como um todo.

## INTRODUCTION

Amongst medical specialties, ultrasonography (US) is a versatile and widely available diagnostic imaging method, being relatively simple to perform, low-cost, non-ionizing and non- or minimally invasive. However, despite the idea of innocuity inherent to the concept of US diagnosis, it is essential to recognize that the sonographic apparatus is a clinical instrument dependent on the physical contact with the patient's body, either inside or outside the hospital environment. Thus, in analogy with the hands of the health professional or with the stethoscope, the sonographic transducer represents an important vector of both cross and nosocomial infections^(1)^. It is known that fomites represented mainly by the conductive gel accumulated on the transducer surface constitute a potential medium of culture and propagation of infectious agents^(2,3)^. Furthermore, in the case of endocavitary examinations, the use of either latex condoms or specific probe covers does not prevent contamination of the transducer by microorganisms, since a rupture of the condom or cover may occur in 2% to 9% of cases^(4,5)^.

Currently, there is no standardization in the practice of cleaning/disinfection of US probes, and a great variety of methods are employed even in the absence of a specific protocol^(6)^.

A survey involving residents and fellows in gynecology and obstetrics revealed that among the 127 individuals who responded to the proposed questionnaire, 83% had never been formally trained to appropriately clean and maintain an US apparatus, and 94% did not know about the existence of a specific protocol for this purpose^(7,8)^. However, despite the availability of international protocols^(9-15)^, it was demonstrated that the current disinfection procedures are not the ideal cleaning method, as suggested by Centers for Disease Control and Prevention (CDC), the North American agency for disease control and prevention^(16,17)^.

Within this context, a concern has been raised about the relevance of cleaning/disinfection of US probes, whether for the purposes of scans involving contact with the skin or endocavitary scans. In the present study, the authors' objective was to review the main concepts regarding this topic, aiming at a better understanding and consequential establishment of strategies for a safe practice without compromising the quality of the examination and the operator productivity.

## INFECTIOUS AGENTS

The infectious agents differ according to the type of examination. In a study evaluating the microbiology of the abdominal skin in 191 pregnant women, with cleaning of the transabdominal transducer at each examination (pre- and post-procedural removal of the conductive gel with a dry cloth), it was observed that 92% of the skin cultures were positive and, in 18% of these cases, potentially pathogenic microorganisms were identified, including *Enterococcus, Staphylococcus aureus, Proteus mirabilis, Escherichia coli*, group B *Streptococcus*, and *Proteus vulgaris*; and in 60% of the cases, the bacteria were transferred from the skin to the transducer^(18)^. Colonies of *Pseudomonas aeruginosa, Klebsiella pneumoniae*, species of *Acinetobacter* and even methicillinresistant *Staphylococcus aureus* have already been isolated in cultures of transabdominal probes^(1,19,20)^. Fungus such as *Candida albicans* were also described^(21)^. Spencer et al. have observed growth of bacteria in 66% of the random swabs of intensively utilized probes^(22)^.

The infectious potential of endocavitary probes (for example, endovaginal and transrectal transducers) is associated with the risk inherent to the direct contact with the mucosas^(4,23)^. In this case, cross and nosocomial infection occurs due the presence of pathogens transmissible through blood or vaginal and rectal secretions. In 3.4% of their cases, Kac et al. have observed the presence of pathogenic flora on the transducers surface after the removal of intact probe covers or condoms. In that study, the following pathogens were identified: *Escherichia coli, Klebsiella pneumoniae, Acinetobacter sp, Acinetobacter lwoffii, Pseudomonas sp, Pseudomonas stutzeri* and *Burkholderia fungorum*^(24)^. Frighteningly, other two studies reported outbreaks of multiresistant *Pseudomonas aeruginosa* associated with the utilization of endocavitary probes in transrectal scans^(25,26)^.

As regards prevalence of viruses in probes, Kac et al. have detected in their cases the presence of viruses - *Epstein Barr* virus (EBV) and human papilloma virus (HPV) - in 1.5% of the transducers after the removal of the condoms or probe covers^(24)^. Casalegno et al. have observed endovaginal transducers contamination with HPV DNA of high oncogenic risk (2.2% contamination risk) even after a disinfection procedure according to the protocol established by the local government^(27)^. The determination of the actual contamination of US probes by viral agents is complicated because of the high prevalence of some viruses (for example, cytomegalovirus, herpes simplex virus, EBV and HPV) or the low frequency of infections by other viruses (for example, human immunodeficiency virus (HIV), hepatitis B virus (HBV), and hepatitis C virus (HCV)). However, the risk for infection is present by simple consideration of the permanence of pathogens on the surface of a transducer utilized for scans in different patients^(16)^.

## CLEANING, STERILIZATION OR DISINFECTION?

Cleaning, which consists in removing any visible residues of either organic or inorganic materials from the transducer surface, can be manually or mechanically performed with running water associated with detergents or enzymatic cleaning products^(17)^. It was demonstrated that the cleaning of the probe with only a paper towel is capable of eliminating up to 45-50% of the pathogenic bacteria. On the other hand, the utilization of physiological saline solution reduces the population of such microorganisms by 76%, and water-and-soap can eliminate up to 98% of the pathogenic bacteria^(6,20,28)^.

Sterilization consists in complete elimination of all forms of microbiotic life, including spores and viruses, utilizing either physical or chemical processes. The main sterilizing agents include: steam pressure vaporization (autoclaving); dry heat; ethylene oxide gas; hydrogen peroxide plasma; and liquid chemicals. Sterilization is the ideal procedure, but the substances and/or the sterilizing process might reduce the durability of the device, being incompatible with the number of scans to be performed and with the protection of both the sonographist and the patient^(17)^.

On the other hand, the concept of disinfection describes the process that eliminates most or all pathogenic microorganisms, excepting bacterial spores, in an inanimate object. Disinfection is divided into three classes, as follows^(8,17)^:

1. Low-level disinfection - Destruction of most bacteria, some viruses and some fungi. *Mycobacterium tuberculosis* or bacterial spores inactivation does not necessarily occur.
2. Intermediate-level disinfection - Inactivation of *Mycobacterium tuberculosis*, bacteria, most viruses and fungi and some bacterial spores.
3. High-level disinfection - Destruction of all microorganisms, except for great amounts of bacterial spores.

In this context, high-level disinfection represents the most appropriate method for guaranteeing the US biosafety. Additionally, it is essential to understand that the disinfection process requires a previous washing of the probe, since the action of the disinfecting substance is more efficacious in the absence of organic or inorganic materials deposited on the transducer surface^(17)^.

## SUBSTANCES AND METHODS: WHICH AND WHEN?

Firstly, it is important to know that the sonographic transducer may be classified and hygienized according the level of infection risk to the patient coming into contact with the ultrasound device during the scan^(29)^. In this case, convex (abdominal), sectorial and linear transducers, for being in contact only with the skin, are considered to be non-critical items due to the low infection risk, since the intact skin provides an effective barrier against microorganisms, and thus the cleaning with detergent or low-level disinfectant is sufficient to guarantee a safe reutilization of the probe^(17,28)^. Differently, the endocavitary probe, due to the risk for contact with (intact) mucosas, is classified as a semi-critical instrument. In such a case, the transducer must be free from any microorganisms (a small number of spores is permissible), and high-level disinfectants should be utilized^(8,17)^. In the case of invasive (critical) examinations, such as punctures and invasion of sterile body cavities, sterilization is mandatory^(17)^.

According to the American Institute of Ultrasound in Medicine (AIUM), the following recommendations should be followed for endocavitary probes disinfection^(8)^:

1. Cleaning - After removing the probe cover (condom, for example), utilize running water and a gauze pad or a soft cloth soaked with liquid soap (dishwashing detergent is the ideal cleaning substance) to remove the residual gel or any other organic/inorganic material from the transducer surface. Consider using a small brush to remove the residues present in grooves or angulations on the probe's design (refer to the manufacturer/user's manual). Subsequently, rinse completely and dry with a soft cloth or paper towel.

2. Disinfection - Immersion of the probe into a high-level disinfection product is most appropriate, but, if immersion is not feasible, the best option is gently scrub the probe with high-level disinfection utilizing a gauze pad or soft cloth. Examples of such disinfectants include: a) 2.4-3.2% glutaraldehyde-based products (Cidex^®^, Metricide^®^); b) non-glutaraldehyde-based products such as ortho-phthalaldehyde 0.55% (Cidex OPA^®^) or hydrogen peroxide and peracetic acid (Cidex PA^®^, Endospor Plus^®^); c) 7.5% hydrogen peroxide (Sporox^®^); d) 5.25% hypochlorite solution (sanitary water - 10 mL in 1 liter of water) - this agent is not recommended by most manufacturers.

The North American health surveillance agency Food and Drug Administration (FDA) provides an online list of approved products for disinfection of medical devices which utilize the above mentioned substances^(30)^. On its turn, the Brazilian Health Surveillance Agency (ANVISA), despite the lack of a specific protocol for US probes, recommends the use of 1.5-2.4% glutaraldehyde, or 0.2-0.32% peracetic acid, or hydrogen peroxide plasma^(31)^. Sonographers should consult the manufacturers about the utilization of any of the mentioned products.

One should consider that not all high-level disinfection products/methods are appropriate for US probes hygienization^(32)^. Transducers are fragile devices containing a sophisticated arrangement of pyezoelectric crystals covered by a receptacle made of plastic and rubber. Therefore, the simple use of abrasive products such as alcohol may damage the probe^(33)^. The use of glutaraldheyde and other aldheydes is questioned because they may damage the health of both physician and patient (skin and mucosal irritation) or impair the clinical procedure itself (for example, damage the gamete or embryo in case of *in vitro* fertilization)^(16,32)^. On its turn, 7.5% hydrogen peroxide solution has been considered to be a very plausible option due to its moderate cost and for neither harming the environment nor the health of physicians and patients^(17)^.

A new option based on the use of ultraviolet C rays has shown to be efficacious to eliminate pathogenic microorganisms^(24)^; however, further studies are required to evaluate the potential harms/benefits of the method. Another available novelty is the Trophon EPR^®^ system, a device where the probe is placed inside and disinfected with hydrogen peroxide steam; besides guaranteeing a high-level disinfection, it is a fast and practical method that is safe for the operator^(34)^.

Finally, it is important to remember that all the probe covers/condom utilized must be tested and approved by the respective regulating institutions. Thus, latex condoms have shown to be more efficient as compared with other specific probe covers^(16)^. For the protection of the patients and staff, it is recommended the use of gloves during all the examinations. Gloves should be used to remove the condom/probe cover as well as to wash the transducer. Subsequently, hands' asepsis is essential to initiate a new scan.

## FINAL CONSIDERATIONS

Notwithstanding the information available in the literature and reviewed in the present study, it is surprising to observe that in the routine US practice few operators perform a rigorous disinfection of probes between scans. Also, few wear gloves during all examinations and habitually wash their hands before and after procedures.

In a recent meta-analysis, Leroy observed that, even after the routine disinfection procedures (low-and intermediate-levels), there was a 12.9% prevalence of pathogenic bacteria and 1% of viruses on the transducer surface. Additionally, this same author observed that the prevalence of infected patients following transrectal US and US-guided biopsy was of 3.1%^(16)^. Thus, it seems reasonable to conclude that the cleaning/disinfection of probes should be systematically and routinely performed, considering that the risk for cross and nosocomial infection is real and is present in the day-by-day sonographers' routine as well as in the patients' lives.

The Heath Technology Faculty of Ribeirão Preto (FATESA), for example, recommends pre- and post-examination handwashing, the use of gloves during the scans, and the use of tissues with specific hydroalcoholic solution for hygienization of US transducers between scans. At the end of each examinations period, the transducers are submitted to cleaning with water and soap, followed by disinfection with 0.2% aqueous chlorhexidine solution. In case of rupture of the protective probe cover/condom during the scanning, washing of the probe with water and soap is performed, followed by immersion into chlorhexidine solution for at least 6 hours. Despite the fact that chlorhexidine is not a high-level disinfectant, it was demonstrated that this substance is effective to eliminate pathogenic microorganisms on transducers' surface^(20)^. Additionally, chlorhexidine is a disinfectant that does not cause damage to the probe, is not expensive and is safe for both the physician and the patient.

In the context of biosafety, it is imperative to assume that the contact with blood and bodily fluids represents a potential source of infection. Thus, with a view on the implementation of practices of cleaning/disinfection, one should understand the infection control principles, take into account the cost-benefit ratio of such measures, and principally consider that they benefit not only the health professional and the patient, but also the society as a whole.
